# Distinct neurostructural, cognitive, and neuropsychiatric associations of plasma p-tau217, and Aβ42/40 in Parkinson’s disease and aging cohorts

**DOI:** 10.3389/fnagi.2026.1854831

**Published:** 2026-06-10

**Authors:** Eleonora Fiorenzato, Simone Cauzzo, Giulia Musso, Roberta Biundo, Chiara Ceolin, Chiara Cosma, Carmelo A. Fogliano, Wassilios G. Meissner, Valentina Misenti, Stefania Moz, Maria Laura Nasi, Francesca Vianello, Martina Berri, Angela Colucci, Renzo Manara, Martina Montagnana, Giuseppe Sergi, Angelo Antonini

**Affiliations:** 1 Neurodegenerative Disease Unit, Department of Neuroscience, University of Padua, Padua, Italy; 2 Department of Medicine–DIMED, University of Padua, Padua, Italy; 3 Laboratory Medicine, University-Hospital of Padua, Padua, Italy; 4 Department of General Psychology, University of Padua, Padua, Italy; 5 IRCCS San Camillo Hospital, Venice, Italy; 6 Department of Neurobiology, Care Sciences and Society, Karolinska Institutet and Stockholm University, Aging Research Center, Stockholm, Sweden; 7 Padova Neuroscience Center (PNC), University of Padua, Padua, Italy; 8 CHU Bordeaux, Service de Neurologie des Maladies Neurodégénératives, IMNc, Place Amélie Raba-Léon, Bordeaux, France; 9 Université de Bordeaux, CNRS, IMN, UMR, Bordeaux, France; 10 Complex Operative Unit (UOC) of the Psychology, Neurology Hospital Division, Padua University Hospital, Padua, Italy; 11 Neuroradiology Unit, DIMED, University of Padua, Padua, Italy

**Keywords:** Alzheimer’s disease co-pathology, amyloid, Lewy body disorders, mild cognitive impairment, MRI, neuroimaging, Parkinson’s disease, ptau217

## Abstract

**Objective:**

Alzheimer’s disease (AD) co-pathology may contribute to cognitive decline and faster progression in Parkinson’s disease (PD). Although blood-based biomarkers enable biological staging along the AD continuum, their contribution in the clinical-biological characterization of PD phenotypes remains unclear. We investigated associations between plasma biomarkers of AD-pathology [phosphorylated-tau-217(p-tau217), amyloid-beta-42/40 (Aβ42/40)], neurodegeneration [neurofilament light chain (NfL)], and neuroinflammation [glial fibrillary acidic protein (GFAP)] with MRI-derived neurostructural indices, cognition, functional independence, and neuropsychiatric symptoms across the PD cognitive spectrum, compared with dementia-free older adults.

**Methods:**

Fifty-eight PD patients and 76 older adults underwent brain MRI, neuropsychological assessment, and plasma biomarker quantification. Multiple linear regressions examined associations between plasma biomarkers and MRI-derived measures (global atrophy, hippocampal volume, AD-specific signature) and clinical measures.

**Results:**

AD-pathology markers (p-tau217 and Aβ42/40) showed stronger associations with neurostructural and clinical/cognitive measures than NfL and GFAP. In both cohorts, higher p-tau217 was associated with AD-like MRI alterations and worse global cognition. Further, in PD, p-tau217 reflected memory and executive dysfunctions, while lower Aβ42/40 was associated with reduced functional independence, visuospatial, and socio-cognitive deficits. In older adults, elevated p-tau217 was linked to subjective cognitive decline, language/memory deficits; lower Aβ42/40 to global atrophy, attention, visuospatial and memory deficits. Neuropsychiatric symptoms in PD (depressive mood, anxiety, apathy) were primarily associated with AD-pathology markers, whereas depressive symptoms in older adults were linked to higher NfL.

**Conclusion:**

Plasma p-tau217 and Aβ42/40 were associated with neurostructural and cognitive impairment in PD and older adults, supporting the potential utility of AD-related plasma biomarkers—particularly p-tau217—for the clinical-biological characterization of cognitive decline in PD.

## 1 Introduction

Blood-based biomarkers are emerging as cost-effective, minimally invasive, and scalable methods that provide critical *in vivo* insights into neurodegenerative processes (Ashton et al., 2020; Alcolea et al., 2023; Campagnolo et al., 2025). Integrating plasma biomarkers reflecting Alzheimer’s disease (AD) pathology, neurodegeneration, and neuroinflammation may help characterizing the multifaceted pathophysiology of Parkinson’s disease (PD), a clinically heterogeneous syndrome with substantial variability in motor and non-motor manifestations, cognitive profiles, disease severity, and progression.

AD co-pathology is relatively common across the spectrum of Lewy body disorders (LBD), including PD and Parkinson’s disease dementia (PDD). Although the severity of the AD neuropathological burden is generally lower in LBD than in AD (Gomperts, 2014), accumulating evidence suggests that concomitant amyloid and tau pathology may contribute to cognitive decline and accelerate disease progression (Smith et al., 2019).

Notably, converging evidence from amyloid PET, CSF, and neuropathological studies has consistently demonstrated that amyloid pathology is common in PD/LBD and contributes to cognitive impairment, particularly affecting memory and attention-executive functions (Fiorenzato et al., 2018; Palermo et al., 2019). Furthermore, amyloid co-pathology has been associated with faster progression to dementia (Akhtar et al., 2016; Buongiorno et al., 2017; Shahid et al., 2019; Myers et al., 2022; Chen et al., 2024) and shorter survival (Jellinger et al., 2002). These findings have strengthened the hypothesis that amyloid- and tau-related mechanisms contribute to the clinical heterogeneity of cognitive decline in LBD.

CSF and PET imaging amyloid biomarkers are the gold standard for detecting AD pathology. However, despite their high diagnostic accuracy, their use may be limited by cost, invasiveness, and restricted accessibility in routine clinical practice. With the advent of anti-amyloid-β (Aβ) treatments for AD, reliable biomarkers to detect AD co-pathology in LBD are needed for patient selection and stratification in clinical trials, as well as for prognostic purposes in clinical care. In this regard, plasma p-tau217 has emerged as a highly sensitive and reliable marker of AD-related pathology, reflecting both brain amyloid accumulation and tau deposition in AD from the preclinical stages of the disease (Ashton et al., 2024; Jack et al., 2024; Malek-Ahmadi et al., 2026). Hitherto, only a limited number of studies have explored the clinical utility of p-tau217 in LBD, and its relevance and application remain largely undefined (Hall et al., 2021; Hannaway et al., 2025; Musso et al., 2025a; Tropea et al., 2026).

To date, studies across the PD spectrum have primarily relied on the measurement of p-tau181 and Aβ42/40 to track AD co-pathology but findings have been inconsistent especially in early PD without dementia (Batzu et al., 2022; Lin et al., 2023; Mizutani et al., 2023; Chen et al., 2026). By contrast, in PDD, elevated p-tau181 levels have been more consistently associated with cognitive impairment (Ye et al., 2025). Furthermore, accurate interpretation of plasma biomarkers is essential to support their reliable use for patient selection and stratification in clinical trials, particularly as emerging therapeutic strategies increasingly target mixed pathological processes (Smith et al., 2026).

Beyond AD-specific biomarkers, neurofilament light chain (NfL) as an index of axonal damage, and glial fibrillary acidic protein (GFAP) reflecting astroglial activation and neuroinflammatory responses have also been widely tested to capture complementary neurodegenerative processes to biologically characterize PD and identify profiles with more severe progression.

Taken together, these findings highlight the need for improved clinico-biological characterization across the PD continuum, ranging from cognitively unimpaired PD to dementia. To address this, we conducted a cross-sectional exploratory study employing a multimodal approach integrating multiple plasma biomarkers with structural MRI indices and detailed clinical characterization to delineate the relative contributions of overlapping pathologies in PD. Specifically, we investigated whether plasma biomarkers reflecting AD-related pathology (p-tau217, Aβ42/40), neurodegeneration (NfL), and neuroinflammation (GFAP) were associated with: (i) MRI-derived measures serving as proxies of early structural neurodegenerative processes (including global atrophy, hippocampal volume and an AD-specific MRI score), (ii) functional independence, and (iii) cognitive performance and neuropsychiatric symptoms. These associations were examined in a cohort of PD patients with varying degree of cognitive impairment and compared with a dementia-free cohort of older adults (age ≥ 65 years).

## 2 Materials and methods

### 2.1 Participants and study design

From the PADUA-CESNE and OPA study cohorts, as part of the “AGE-it” project on normal aging^1^, a total of 134 participants were recruited at the Neurodegenerative Disease Unit of the Padua University Hospital (Italy), from November 2023 to December 2025. The total cohort includes 58 PD patients and 76 older adults.

**FIGURE 1:**
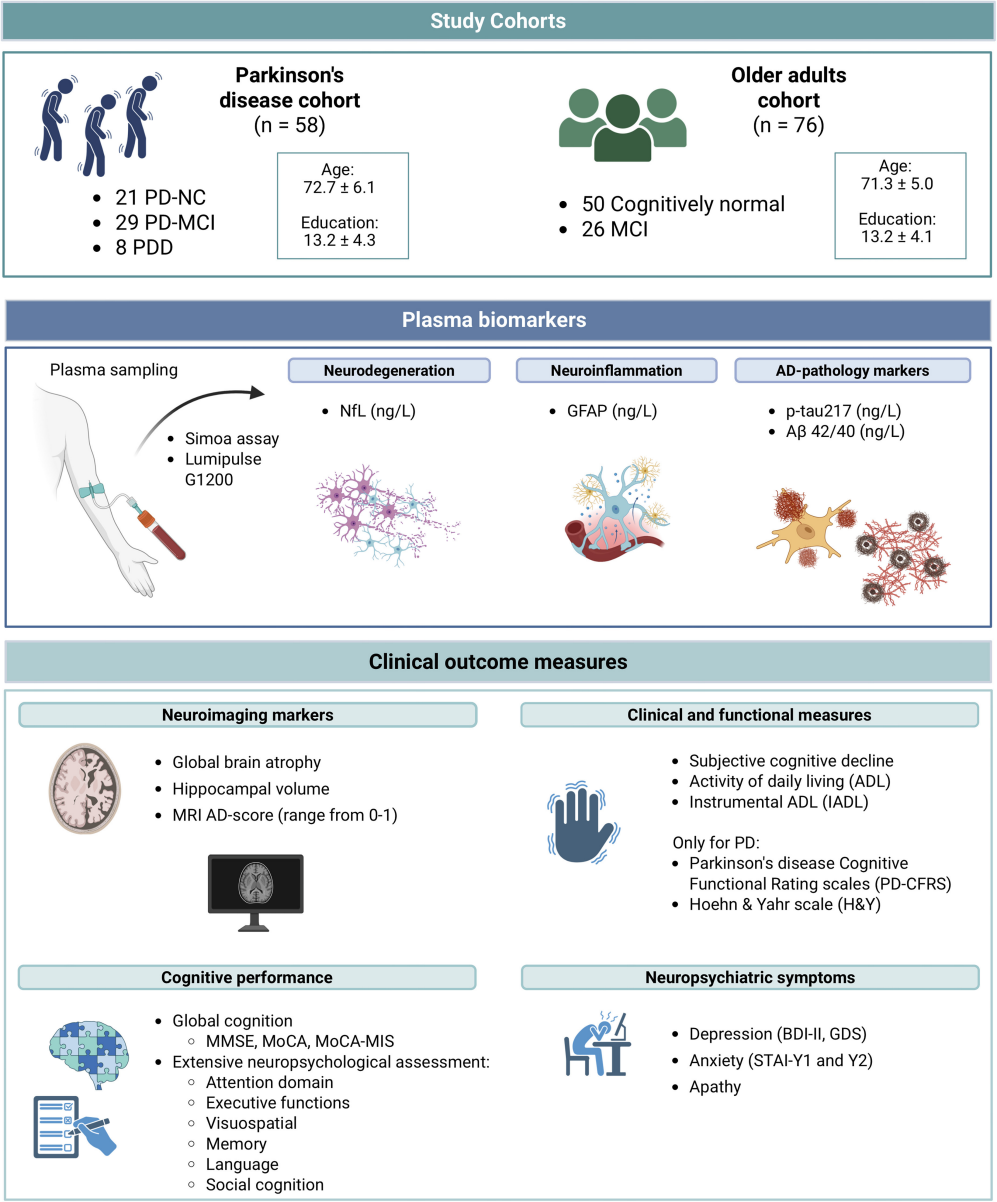
Study design overview: cohort characterization, plasma biomarker profiling, and clinical outcome assessments.

The multimodal study protocol comprised brain MRI, clinical, neuropsychological/behavioral assessments, and blood sampling to analyze plasma biomarkers. An overview of study and participants recruitment are displayed in Figure 1.

PD with normal cognition (PD-NC), with mild cognitive impairment (MCI; PD-MCI) and dementia (PDD), were classified based on a Level-II cognitive assessment, as previously described (Fiorenzato et al., 2024b). PDD subjects were in the early stages of dementia to ensure compliance with all study procedures. PD diagnoses were established by movement disorder specialists. Motor severity was staged using the Hoehn and Yahr (H&Y) scale, and antiparkinsonian treatment was standardized by calculating the levodopa and the dopamine agonist equivalent daily dosages for each patient (Jost et al., 2023).

The 76 community-based older adults (age ≥ 65 years) were recruited via media and community advertisements. Exclusion criteria included the presence of MRI abnormalities [such as cerebral vascular lesions, diffuse white matter hyperintensities (Fazekas = 3), tumors or other space-occupying lesions], a history of head injuries, diagnosis of dementia, and any significant psychiatric, neurological, or systemic comorbidities. One informant for each participant was also enrolled in the study, providing collateral information on participant activities of daily living (ADL) and cognitive functioning. Presence of MCI was determined based on the cognitive assessment, as detailed below. Written informed consent was obtained from all participants in accordance with the Declaration of Helsinki, and the study was approved by the Clinical Research Ethics Committee of Padua (5707/AO/23).

### 2.2 Neuropsychological and behavioral evaluation

Participants underwent a comprehensive cognitive assessment, as previously described (Musso et al., 2025a). For further details see Supplementary Material.

Briefly, global cognition was evaluated using the Mini-Mental Scale Examination (MMSE) and Montreal Cognitive Assessment (MoCA), with scores adjusted for age and education, based on Italian normative data (Fiorenzato et al., 2024a; Dapor et al., 2025). The MoCA Memory Index Score (MoCA-MIS) was derived from the delayed memory subtest to evaluate semantic cueing efficacy during retrieval, with lower scores specifically reflecting amnestic-type encoding deficits (Julayanont et al., 2014).

The cognitive assessment further explored six cognitive domains: memory, executive functions, attention, visuospatial abilities, language, and social cognition. Performance on each test was standardized by computing the z-scores using Italian norms; then, domain-specific composite z-scores were calculated as the arithmetic mean of the constituent tests. Based on this assessment, PD patients were classified as PD-NC (*n* = 21), PD-MCI (*n* = 29), or early PDD (*n* = 8).

Similarly, for the older adult cohort, MCI was diagnosed according to DSM-5 criteria and updated guidelines (Dunne et al., 2021), requiring: i) subjective cognitive decline, reported by the participant or an informant; ii) objective cognitive impairment in at least one domain, defined as performance 1.5 standard deviations below the normative mean; and iii) preserved independence in ADL.

Following these criteria, 26 older adults were classified as MCI, and 50 as cognitively unimpaired (CU).

Regarding subjective cognitive decline in the older adult cohort, the Multidimensional Assessment of Subjective Cognitive Decline (MASCoD) scale was administered, with scores above 8 indicating significant cognitive complaints (Maffoni et al., 2022). In both cohorts, functional independence was evaluated using the ADL and Instrumental ADL (IADL) scales. In PD, the impact of cognitive dysfunctions on daily functioning was assessed with the PD Cognitive Functional Rating Scale (PD-CFRS) (Garon et al., 2024).

Finally, neuropsychiatric symptoms were screened: depressive symptoms were assessed using the Beck’s Depression Inventory (BDI-II) and the Geriatric Depression Scale (GDS); apathy was evaluated with the Starkstein Apathy Scale, and anxiety with the State-Trait Anxiety Inventory (STAI-Y1 and Y2) (Aiello et al., 2022).

### 2.3 Assessment of plasma biomarkers

Concentrations of NfL, GFAP, and Aβ42/40 were measured in K-2 ethylenediaminetetraacetic acid (EDTA) plasma samples using a research- use-only (RUO) Simoa assays on a semi-automated SR-X platform (NfL and GFAP: Neuro 2-Plex B Advantage kit; Aβ42/40: Neuro 3-Plex A Advantage kit) (Quanterix, United States). After centrifugation, samples were aliquoted into polypropylene tubes and stored at −80°C. Prior to analysis, samples were thawed at room temperature for at least 30 min and centrifuged at 10,000 g for 5 min. Analytical variability was monitored by including two manufacturer-provided quality control levels (low and high) in each run; the coefficient of variation was < 20% for both levels. Instead, p-tau217 was quantified in K-2 EDTA plasma samples using the RUO Lumipulse G1200 assay (Fujirebio, Japan), as previously published (Musso et al., 2025b).

### 2.4 Neuroimaging data

Structural T1-weighted and FLAIR images were acquired on a 3T scanner and processed via FreeSurfer (v6.0.0).^2^ For more details, see Supplementary Materials. Three primary MRI indices were derived: (i) hippocampal volume (adjusted for age and intracranial volume), (ii) global brain atrophy (Orellana et al., 2016), and (iii) a machine-learning-derived AD-specific MRI score (with a range from 0 to 1). This AD score (BAAD software) (Ishida et al., 2021) utilizes a support vector machine trained on the ADNI dataset to estimate AD likelihood based on whole-brain and medial temporal structures—including the entorhinal cortex, hippocampus, and amygdala (Shiino et al., 2021; Syaifullah et al., 2021).

### 2.5 Statistical analyses

Data normality and homogeneity of variance were assessed using the Shapiro–Wilk and Levene’s tests, respectively. Categorical variables were evaluated using Chi-square tests. For continuous variables, group differences were analyzed based on data distribution: normally distributed data were compared using Student’s *t*-tests or analysis of variance (ANOVA), followed by Tukey’s *post hoc* test, whereas non-normally distributed data were analyzed by Mann-Whitney *U* test for direct comparisons between MCI vs. CU groups or the Kruskal-Wallis test, supplemented by Dwass-Steel-Critchlow-Fligner for pairwise comparisons. Differences in plasma biomarkers across groups (PD subgroups vs. CU and MCI) were evaluated using Quade’s non-parametric ANCOVA, incorporating age as a covariate to control for potential confounding effects.

All participants had a complete set of plasma biomarkers (NfL, GFAP, p-tau217, Aβ42/40 ratio). Associations between log-transformed biomarker levels and clinical outcomes were modeled using multiple linear regression, followed by backward feature selection. These outcomes included: (i) MRI features (i.e., global brain atrophy, hippocampal volume, and MRI AD-score); (ii) functional measures; (iii) cognitive performance (MoCA, MMSE, and domain-specific composite z-scores); and (iv) neuropsychiatric symptoms. Given the exploratory design of the study and the relatively small sample size, regression analyses were performed to investigate associations between plasma biomarkers and clinical outcomes across different domains. No additional correction for multiple comparisons was applied to these exploratory regression analyses. Results were reported as standardized coefficients (β) with 95% confidence intervals to ensure comparability across models. Age was included as a covariate in all models, except in cases where the independent variable was already age-adjusted. Supplementary analyses additionally controlling for H&Y stage are presented in the Supplementary Materials. To ensure model integrity, multicollinearity was assessed using tolerance and variance inflation factor indices; all variables in regression models had a variance inflation factor < 5 and a tolerance > 0.2, which are not deemed to indicate problematic levels of multicollinearity (O’brien, 2007). Partial Spearman’s correlations between plasma markers were calculated (Supplementary Materials), followed by False Discovery Rate (FDR) correction was applied for multiple comparison corrections. All the statistical analyses were performed using R (version 4.2.3) and IBM SPSS Statistics (version 24); the statistical significance threshold was set at *p* ≤ 0.05.

## 3 Results

### 3.1 Participants demographic and clinical characteristics

PD subgroups did not differ in terms of age, education, sex, disease duration, or antiparkinsonian treatment (Table 1). However, PDD patients exhibited greater motor severity compared with PD-NC, as reflected by higher H&Y stage (*MD* = –0.73, *p* = 0.012). Plasma p-tau217 levels were significantly higher in both PD-MCI and PDD compared to PD-NC (*MD* = –3.70, *p* = 0.001; and *MD* = –2.63, *p* = 0.029, respectively), whereas no significant group differences were observed for the other plasma biomarkers. Regarding neurostructural measures, PDD showed a higher MRI-derived AD score compared with PD-NC (*W* = 4.00, *p* = 0.013). No significant differences were found in global brain atrophy (*p* = 0.620), although hippocampal volume showed a trend toward significance (*p* = 0.061)

**TABLE 1:** Parkinson’s disease cohort (*N* = 58), demographical, plasma markers and MRI characteristics.

		PD-NC (*n* = 21)	PD-MCI (*n* = 29)	PDD (*n* = 8)	*p*-value	*Post hoc*
Age		**71.57 (5.46)**	**73.28 (7.08)**	**73.75 (3.11)**	**0.552**	
Sex (f/m)		10/11	12/17	2/6	0.543	
Education (years)		14.62 (3.6)	12.03 (4.51)	13.38 (4.14)	0.103	
Disease duration	§	7.29 (5.46)	8.69 (6.2)	6.50 (4.54)	0.595	
H&Y (ON state)		1.83 (0.6)	2.14 (0.53)	2.56 (0.78)	**0.014**	PDD > PD-NC
LEDD	§	528.83 (447.91)	474.93 (278.88)	557.13 (442.25)	0.973	
DAED	§	89.62 (98.14)	43.47 (71.19)	19.63 (39.00)	0.06	
Blood-based markers						
NfL (ng/L)	§	21.52 (20.8)	19.09 (7.33)	24.5 (17.21)	0.619	
GFAP (ng/L)	§	272.49 (293.5)	213.53 (97.37)	267.96 (136.18)	0.537	
Aβ42/40 (ng/L)	§	0.04 (0.01)	0.05 (0.04)	0.04 (0.03)	0.520	
p-tau217 (ng/L)	§	0.09 (0.04)	0.17 (0.12)	0.17 (0.11)	**< 0.001**	PD-NC < PD-MCI/PDD
MRI markers						
Global atrophy index	§	30.16 (13.04)	29.71 (7.65)	26.79 (12.79)	0.62	
Hippocampal volume (adj)		6635.48 (681.98)	6152.65 (783.47)	6240.71 (366.07)	0.061	
MRI AD-score	§	0.18 (0.21)	0.3 (0.28)	0.56 (0.29)	**0.006**	PD-NC < PDD

Mean and standard deviation are displayed. Significant *p*-values are shown in bold. §, not normally distributed variables. PD, Parkinson’s disease; PD-NC, PD with normal cognition; PD-MCI, PD with mild cognitive impairment; PDD, PD with dementia; H&Y, Hoehn and Yahr Scale; LEDD, levodopa equivalent daily dose; DAED, dopamine agonist equivalent daily dose; NfL, neurofilament light chain; GFAP, glial fibrillary acidic protein; Aβ42/40, amyloid-beta-42/40; p-tau217, phosphorylated-tau-217; AD, Alzheimer’s disease. *Post hoc* significant differences after multiple comparison corrections are displayed.

As reported in Table 2, PDD patients performed worse than both PD-NC and PD-MCI on the MoCA and MMSE (all *p* < 0.005). A trend toward lower MoCA performance was also observed in PD-MCI compared with PD-NC (*W* = –3.29, *p* = 0.052). In addition, PDD showed poorer memory-encoding abilities than PD-NC, as indicated by lower MoCA-MIS scores (*W* = –3.32, *p* = 0.049). Functional independence (IADL and PD-CFRS) was significantly reduced in PDD compared with both PD-NC and PD-MCI (all *p* < 0.001).

**TABLE 2:** Parkinson’s disease cohort (*N* = 58) cognitive, functional and behavioral characteristics.

		PD-NC (*n* = 21)	PD-MCI (*n* = 29)	PDD (*n* = 8)	*p*-value	*Post hoc*
Global cognition						
MMSE	§	27.75 (2.09)	25.99 (2.44)	21.49 (2.78)	**< 0.001**	PDD < PD-NC/PD-MCI
MoCA	§	26.28 (1.93)	24.05 (3.7)	17.06 (3.94)	**< 0.001**	PDD < PD-NC/PD-MCI
MoCA-MIS	§	11.29 (2.85)	10.32 (3.45)	8.05 (3.57)	**0.042**	PDD < PD-NC
Functional independence						
PD-CFRS	§	1.14 (1.11)	2.29 (2.36)	15.75 (5.04)	**< 0.001**	PDD < PD-NC/PD-MCI
ADL	§	5.67 (0.48)	5.66 (0.55)	4.13 (2.36)	0.274	–
IADL	§	6.43 (1.43)	5.9 (1.59)	2.5 (1.07)	**< 0.001**	PDD < PD-NC/PD-MCI
Neuropsychological assessment (z-compound)						
Attention	§	0.03 (0.5)	−0.52 (0.96)	−4.03 (1.38)	**< 0.001**	PDD < PD-NC/PD-MCI
Executive Functions	§	0.36 (0.29)	−0.32 (0.66)	−2.69 (1.49)	**< 0.001**	PDD < PD-MCI < PD-NC
Visuospatial	§	−0.38 (0.61)	−1.11 (1.28)	−4.06 (2.43)	**< 0.001**	PDD < PD-MCI < PD-NC
Memory		−0.1 (0.66)	−1.13 (0.86)	−2.39 (1.28)	**< 0.001**	PDD < PD-MCI < PD-NC
Language		0.19 (0.69)	−0.83 (0.87)	−2.40 (0.53)	**< 0.001**	PDD < PD-MCI < PD-NC
Social Cognition		0.4 (0.68)	−0.57 (1.26)	−3.00 (1.6)	**< 0.001**	PDD < PD-MCI < PD-NC
Behavioral assessment						
BDI-II	§	9.38 (8.56)	12.61 (7.66)	24.17 (10.46)	**0.008**	PDD > PD-NC/PD-MCI
GDS		8.22 (6.74)	10.5 (5.63)	14.25 (3.78)	0.105	–
STAI-Y1	§	35.62 (10.82)	41.84 (10.25)	54.67 (11.24)	**0.002**	PDD > PD-NC/PD-MCI
STAI-Y2		37.43 (11.35)	43.89 (7.42)	52.17 (9)	**0.017**	PDD > PD-NC/PD-MCI
Apathy		11.76 (4.07)	14.83 (4.74)	20.67 (6.09)	**0.009**	PDD > PD-NC/PD-MCI

Mean and standard deviation are displayed. Significant *p*-values are shown in bold. §, not normally distributed variables. PD, Parkinson’s disease; PD-NC, PD with normal cognition; PD-MCI, PD with mild cognitive impairment; PDD, PD with dementia; MMSE, Mini-Mental Scale Examination; MoCA, Montreal Cognitive Assessment; MoCA-MIS, MoCA- Memory Index Score; PD-CFRS, Parkinson’s Disease Cognitive Functional Rating Scale; ADL, Activities of Daily Living; IADL, instrumental ADL; BDI-II, Beck’s Depression Inventory; GDS, Geriatric Depression Scale; STAI-Y1, State-Anxiety Inventory, STAI-Y2, Trait-Anxiety Inventory.

Across neuropsychological domains, performance progressively worsened from PD-NC to PD-MCI to PDD, indicating a gradient of impairment. Finally, the behavioral assessment revealed significantly higher levels of depressive symptoms, apathy, and anxiety in PDD compared with both PD-MCI and PD-NC (all *p* < 0.05).

Regarding the older adult cohort, the MCI group was significantly older than CU (*p* = 0.002) and had a lower educational level (*p* < 0.001), while no differences emerged in sex distribution (Table 3). In terms of plasma biomarkers, individuals with MCI showed significantly higher p-tau217 levels and a lower Aβ42/40 ratio compared to CU individuals (both *p* = 0.038), whereas no significant group differences were observed for the other plasma markers. No significant differences were detected between groups in the investigated MRI-derived features. Clinically, MCI participants reported greater subjective cognitive impairments (*p* = 0.008) and showed poorer global cognitive performance, as reflected by lower MMSE scores (*p* = 0.040). Moreover, MCI individuals performed worse on the MoCA memory index (MoCA-MIS; *p* = 0.007). Across neuropsychological testing, the MCI group exhibited significantly poorer performance in all examined cognitive domains compared to CU. Conversely, no significant differences were found in functional independence or behavioral symptoms.

**TABLE 3:** Older adult cohort (*N* = 76) characteristics.

		CU (*n* = 50)	MCI (*n* = 26)	Statistics	*p*-value
Age		70.02 (3.8)	73.77 (6.22)	−3.26 (74)	**0.002**
Sex (f/m)		24/26	12/14	0.023 (1,76)	0.878
Education (years)	§	14.82 (3.65)	11.27 (3.95)	334.50 (74)	**< 0.001**
Plasma markers					
NfL (ng/L)	§	12.76 (5.52)	15.45 (7.16)	1.75 (1,73)	0.190
GFAP (ng/L)	§	181.77 (89.53)	203.52 (100.72)	0.22 (1,73)	0.642
Aβ42/40 (ng/L)	§	0.05 (0.01)	0.04 (0.01)	4.45 (1,73)	**0.038**
p-tau217 (ng/L)	§	0.13 (0.17)	0.22 (0.24)	4.48 (1,73)	**0.038**
MRI markers					
Global atrophy index		51.33 (20.66)	41.97 (19.59)	1.96 (74)	0.061
Hippocampal volume (adjusted)		6426.24 (558.84)	6240.48 (785.79)	1.19 (74)	0.237
MRI AD-score	§	0.13 (0.18)	0.16 (0.23)	611.00 (74)	0.673
Global cognition					
MASCoD	§	5.56 (2.92)	7.35 (3.29)	410.5 (74)	**0.008**
MMSE	§	27.72 (2.12)	26.54 (2.21)	466 (74)	**0.04**
MoCA		25.91 (2.38)	24.63 (3.45)	1.9 (74)	0.061
MoCA-MIS	§	11.87 (2.7)	10.11 (2.76)	401 (74)	**0.007**
Functional independence					
ADL	§	5.98 (0.14)	5.89 (0.33)	588 (74)	0.082
IADL	§	6.44 (1.51)	6.35 (1.57)	625 (74)	0.758
Neuropsychological assessment (z-compound)					
Attention		0.24 (0.57)	−0.26 (0.56)	3.64 (74)	**< 0.001**
Executive functions	§	0.29 (0.83)	−0.17 (0.89)	454 (74)	**0.032**
Visuospatial		0.13 (0.66)	−0.71 (0.85)	4.76 (74)	**< 0.001**
Memory		0.11 (0.96)	−1.14 (1.05)	5.21 (74)	**< 0.001**
Language		0.61 (0.91)	−0.64 (1.13)	5.20 (74)	**< 0.001**
Social cognition		0.35 (0.72)	−0.52 (1)	4.33 (74)	**< 0.001**
Behavioral assessment					
BDI-II	§	5.36 (4.07)	7.15 (4.9)	514.5 (74)	0.138
GDS	§	4.44 (4.12)	5.54 (5.46)	594 (74)	0.541
STAI-Y1	§	32.04 (6.58)	34.27 (7.91)	506 (74)	0.115
STAI-Y2		36.52 (7.32)	38.04 (7.37)	−0.86 (74)	0.395
Apathy	§	11.8 (4.06)	13.69 (4.15)	487 (74)	0.074

Mean and standard deviation are displayed. Significant *p*-values are shown in bold. §, not normally distributed variables. CU, cognitive unimpaired participants; MCI, mild cognitive impairment; NfL, neurofilament light chain; GFAP, glial fibrillary acidic protein; Aβ42/40, amyloid-beta-42/40; p-tau217, phosphorylated-tau-217; AD, Alzheimer’s disease; MASCoD, Multidimensional Assessment of Subjective Cognitive Decline; MMSE, Mini-Mental Scale Examination; MoCA, Montreal Cognitive Assessment; MoCA-MIS, MoCA- Memory Index Score; PD-CFRS, Parkinson’s Disease Cognitive Functional Rating Scale; ADL, Activities of Daily Living; IADL, instrumental ADL; BDI-II, Beck’s Depression Inventory; GDS, Geriatric Depression Scale; STAI-Y1, State-Anxiety Inventory, STAI-Y2, Trait-Anxiety Inventory.

### 3.2 Association between plasma biomarkers and neurostructural indices

**FIGURE 2:**
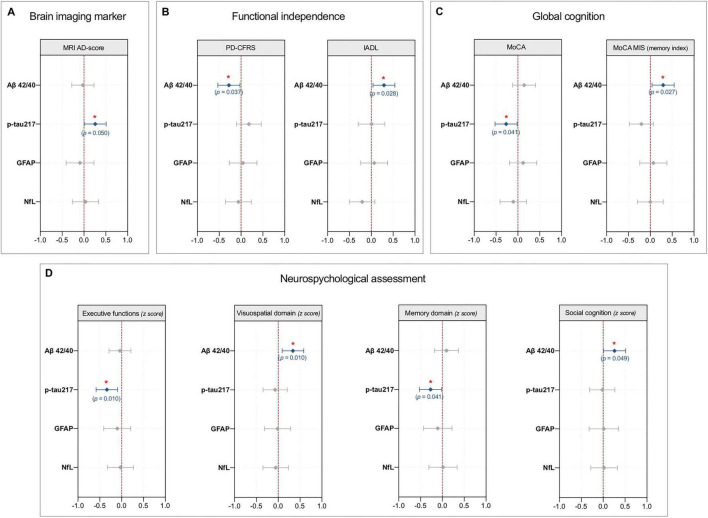
Associations between plasma biomarkers and clinical/imaging markers in Parkinson’s disease (*N* = 58). Forest plots display associations with **(A)** neuroimaging indexes, **(B)** functional measures, and **(C,D)** cognitive domains. Results are derived from Multiple Linear Regression models, followed by backward feature selection. Data points represent standardized regression coefficients (β) with 95% confidence intervals (CIs), indicating the change in the dependent variable per log unit increase in biomarker concentration.

In the PD cohort, multiple linear regression showed that higher plasma p-tau217 levels were associated with a greater MRI AD-signature (β = 0.26, *t* = 2.00, *p* = 0.050; Figure 2A). In contrast, no significant associations were observed between the other plasma biomarkers (NfL, GFAP, or Aβ42/40 ratio) and the other neurostructural indices, including global brain atrophy and hippocampal volume.

**FIGURE 3:**
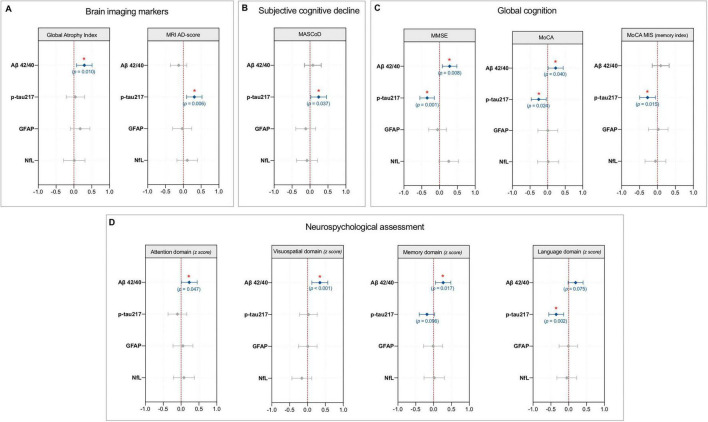
Associations between plasma biomarkers and clinical/imaging markers in the older adult cohort (*N* = 76). Forest plots display associations with **(A)** neuroimaging indexes, **(B)** Subjective Cognitive Decline (SCD) measures, and **(C,D)** cognitive domains. Results are derived from Multiple Linear Regression models, followed by backward feature selection. Data points represent standardized regression coefficients (β) with 95% confidence intervals (CIs), indicating the change in the dependent variable per log unit increase in biomarker concentration.

In the cohort comprising CU/MCI individuals, distinct pattern of associations emerged (Figure 3A). Lower Aβ42/40 ratios (indicative of higher brain amyloid burden) were associated with greater global atrophy (β = 0.29, *t* = 2.64, *p* = 0.010) and the MRI AD-score was positively linked to plasma p-tau217 levels (β = 0.31, *t* = 2.82, *p* = 0.006). No other significant relationships were identified for hippocampal volume.

### 3.3 Association between plasma biomarkers and functional and clinical measures

The associations between plasma biomarkers and functional measures in PD are presented in Figure 2B. Lower plasma Aβ42/40 ratios were significantly associated with greater functional cognitive impairment, as assessed by the PD-CFRS (β = –0.28, *t* = –2.14, *p* = 0.037), and with reduced functional independence on the IADL scale (β = 0.29, *t* = 2.26, *p* = 0.028). No significant associations were observed between plasma markers and motor severity (H&Y).

In the older adult group, plasma biomarker levels were not significantly associated with functional outcomes, as measured by ADL and IADL.

### 3.4 Association between plasma biomarkers and cognitive performance

The relationship between plasma biomarkers and cognition in the PD cohort is summarized in Figures 2C,D. Regarding global cognition, higher p-tau217 levels were associated with lower MoCA scores (β = –0.27, *t* = –2.09, *p* = 0.041), while a lower Aβ42/40 ratio was associated with poorer performance on its memory index (MoCA-MIS; β = 0.29, *t =* 2.27, *p* = 0.027). No associations were found between plasma biomarkers and MMSE scores.

Analysis of cognitive domains (expressed as z-scores) revealed a negative association between p-tau217 levels and executive functions (β = –0.33, *t =* –2.68, *p* = 0.010); in addition, higher p-tau217 levels were associated with increase memory deficits (β = –0.27, *t =* –2.09, *p* = 0.041).

Conversely, a lower Aβ42/40 ratio was linked to worse visuospatial abilities (β = –0.33, *t* = 2.67, *p* = 0.010) and poorer socio-cognitive abilities (β = –0.26, *t =* 2.01, *p* = 0.049).

In older adults, subjective cognitive decline, as specifically assessed by MASCoD scale, was associated with higher plasma p-tau217 levels (β = –0.24, *t =* 2.13, *p* = 0.037; Figure 3B).

For global cognition (Figure 3C), higher p-tau217 (β = –0.35, *t =* –3.35, *p* = 0.001) and lower Aβ42/40 ratios (β = –0.28, *t =* 2.72, *p* = 0.008) were both significant predictors of lower MMSE scores. Similarly, MoCA performances was predicted by both lower Aβ42/40 ratios (β = 0.23, *t* = 2.09, *p* = 0.040) and higher p-tau217 (β = –0.25, *t =* –2.31, *p* = 0.024). Furthermore, p-tau217 was negatively correlated with MoCA-MIS (β = –0.28, *t =* –2.48, *p* = 0.015), indicating that higher p-tau217 levels specifically related to memory-encoding deficits.

Domain-specific analyses showed that higher Aβ42/40 ratio was positively associated with better performance in the attention (β = 0.23, *t =* 2.02, *p* = 0.047), visuospatial function (β = 0.39, *t =* 3.66, *p* < 0.001), and memory (β = 0.27, *t =* 2.44, *p* = 0.017) (Figure 3D). Within the memory model, p-tau217 was also retained as a contributing factor, though it did not reach significance (*p* = 0.096).

Finally, poorer language abilities were significantly predicted by elevated p-tau217 (β = –0.35, *t =* –3.29, *p* = 0.002), with the Aβ42/40 ratio remaining in the model as a non-significant contributor (*p* = 0.075).

### 3.5 Association between plasma biomarkers and neuropsychiatric symptoms

**FIGURE 4:**
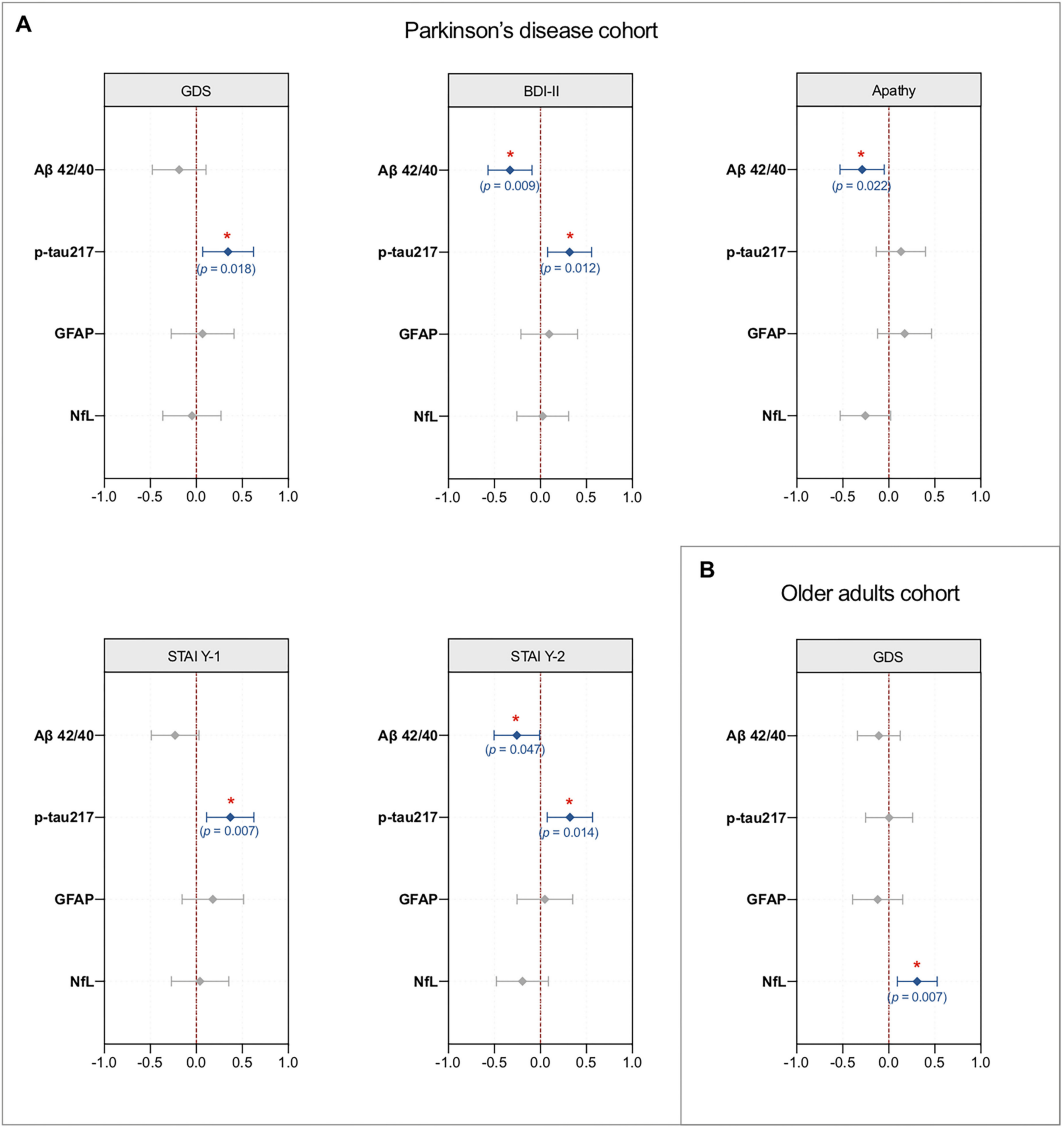
Associations of plasma biomarkers with neuropsychiatric symptoms in Parkinson’s disease patients (*N* = 58) **(A)** and older adult cohort (*N* = 76) **(B)**. Significant results from Multiple Linear Regression, followed by backward feature selection. Standardized regression coefficients (β) and 95% confidence intervals (CIs) are displayed, indicating the change in the dependent variable per log unit increase in biomarker concentration.

The associations between plasma biomarkers and neuropsychiatric symptoms in PD are displayed in Figure 4A. Higher p-tau217 levels were significantly associated with increased depressive symptoms, as assessed by the GDS (β = 0.35, *t =* 2.45, *p* = 0.018) and the BDI-II scale [higher p-tau217 (β = 0.32, *t =* 2.60, *p* = 0.012) and lower Aβ42/40 ratio (β = –0.33, *t =* –2.70, *p* = 0.009)]. Apathic symptoms were also primarily associated with a lower Aβ42/40 ratio (β = –0.29, *t =* –2.36, *p* = 0.022).

Regarding anxiety, higher state anxiety was associated with elevated p-tau217 (β = 0.37, *t =* 2.82, *p* = 0.007), whereas trait anxiety was primarily predicted by higher p-tau217 (β = 0.32, *t =* 2.55, *p* = 0.014) and a lower Aβ42/40 ratio (β = –0.26, *t =* –2.03, *p* = 0.047).

In contrast, in the older adult cohort (Figure 4B), higher NfL levels were associated with increased depressive symptoms on the GDS (β = 0.31, *t =* 2.80, *p* = 0.007). No significant associations were observed between plasma biomarkers and anxiety or apathy symptoms in this group.

## 4 Discussion

In the present cross-sectional exploratory study, we examined the association between several plasma biomarkers (NfL, GFAP, p-tau217, Aβ42/40) and MRI-derived neurostructural indices, functional/clinical measures, cognitive performance, and neuropsychiatric symptoms across clinical-cognitive groups. In both PD and older adult cohorts, AD-related plasma markers—p-tau217, and the Aβ42/40 ratio—emerged as the main biological correlates of neurostructural and clinical status, whereas NfL and GFAP did not show significant contributions.

### 4.1 MRI-derived neurostructural indices

One of the main findings of this study is the observed association between higher plasma p-tau217 and AD-specific MRI signatures in both PD and aging cohorts. Previous work has demonstrated the high accuracy of this MRI-derived AD score in identifying amyloid-positive individuals with MCI (Shiino et al., 2021; Syaifullah et al., 2021). Thus, our results extend this finding by supporting p-tau217 as a sensitive marker of AD-related neurodegeneration not only in aging and MCI, but potentially also in PD.

Consistent with this interpretation, plasma p-tau217 showed greater sensitivity in differentiating cognitive subgroups across both cohorts. P-tau217 levels were more elevated in cognitively impaired PD groups (PD-MCI and PDD vs. PD-NC) and in MCI compared with CU individuals, whereas the Aβ42/40 ratio appeared sensitive only in the older adult cohort, but not in PD.

Indeed, the superior diagnostic and prognostic performance of p-tau217 has been widely reported (Ashton and Zetterberg, 2025; Thal et al., 2025), and in longitudinal studies, only p-tau217 has shown stable increases associated with clinical deterioration and brain atrophy compared with other markers, including Aβ42/40 or p-tau181, even in preclinical AD stages (Ashton et al., 2022). Our findings are consistent with previous studies linking higher p-tau217 levels to reduced cortical thickness in AD-signature regions in dementia-free adult cohorts (Mattsson-Carlgren et al., 2020; Zurrón et al., 2026). Importantly, we extend these observations to PD, where elevated p-tau217 showed associations with more pronounced AD-like neurostructural changes and greater cognitive impairment, supporting a possible relationship between AD plasma biomarkers and AD-like neurostructural changes in PD.

Although this AD-specific MRI index appeared more sensitive than hippocampal or global atrophy measures alone, it is worth noting that it was originally developed within the AD framework and may not fully capture the complexity of PD-related neurodegeneration (Shiino et al., 2021; Syaifullah et al., 2021). Therefore, further studies are needed to confirm our findings and develop a more PD-specific neurostructural index potentially associated with AD-related pathology.

In the older adult cohort, lower plasma Aβ42/40 ratios—reflecting a greater cerebral amyloid burden—were associated with increased global brain atrophy, in line with models of AD neurodegeneration in which amyloid accumulation precedes widespread cortical atrophy (Pieperhoff et al., 2025). By contrast, this association was not observed in the PD cohort, despite greater global atrophy in PD subgroups compared with CU. Instead, structural changes in PD were specifically related to p-tau217 and confined to AD-signature regions, suggesting that amyloid may act synergistically with tau to drive focal cortical thinning in vulnerable networks. Conversely, the global atrophy observed in PD may reflect α-synuclein-related neurodegeneration occurring independently of the amyloid cascade (Garon et al., 2021; Labrador-Espinosa et al., 2023).

Notably, NfL, a plasma marker of neurodegeneration, did not capture global cortical atrophy in the PD cohort. This lack of association may reflect the complex and multifactorial nature of neurodegeneration in PD, especially if patients of different cognitive severity are considered. Indeed, many studies reporting correlations between NfL and GM/WM integrity measures have focused primarily on early-stage PD cohorts without cognitive impairment (Gallingani et al., 2024). We can surmise that as PD progresses across the cognitive spectrum, neurostructural changes reflect multiple overlapping mechanisms, with axonal damage representing only one component of the broader degenerative process.

Finally, hippocampal volume alone did not significantly differ across clinical-cognitive groups, suggesting that composite indices such as the MRI-derived AD score, which integrate hippocampal volume with additional cortical regions, provides greater sensitivity in detecting AD-related neurodegenerative patterns.

### 4.2 Functional independence and motor severity

Beyond neurostructural measures, a lower plasma Aβ42/40 ratio emerged as the strongest predictor of reduced instrumental functional independence in PD, as assessed by the IADL scale. Lower Aβ42/40 was also associated with greater cognitive-related functional disability, measured by the PD-CFRS. Although evidence directly linking amyloid burden to IADL dysfunction in PD remains limited, our findings align with studies in non-PD populations showing that amyloid pathology is associated with functional decline across the AD spectrum (Jutten et al., 2019).

In contrast, we found no significant associations between plasma biomarkers and functional independence in our older adult cohort, assessed by ADL and IADL scales. This may reflect a lack of sensitivity of these scales for mild, although significant, cognitive alterations. Regarding motor severity in the PD cohort, none of the plasma biomarkers were significantly associated with a more advanced disease stage as measured by the H&Y scale. Although the literature is heterogeneous, with some studies reporting associations between NfL and GFAP and motor severity (Lin et al., 2019; Yin et al., 2022; Pilotto et al., 2024), our results align with other works that did not identify such relationship (Batzu et al., 2022; Hiraga et al., 2024). This discrepancy may partly reflect the role of NfL, a marker of axonal damage, as a more sensitive indicator of longitudinal disease progression than of cross-sectional motor severity (Pilotto et al., 2024).

### 4.3 Cognitive performance

Our findings indicate that distinct AD-related biomarkers (p-tau217 and Aβ42/40) were associated with different patterns of cognitive impairment in PD versus typical aging/MCI.

Within the PD cohort, plasma p-tau217 emerged as a primary determinant of global cognition (MoCA), executive function, and memory. Higher p-tau217 concentrations were associated with cognitive domains that are among the most frequently impaired in PD (Lawrence et al., 2016). Indeed, executive function and verbal memory have been highlighted as critical domains in PD, as impairments in these functions predict subsequent progression to dementia (Levy et al., 2002). Thus, our results suggest that AD-related pathology, reflected by elevated p-tau217, may substantially contribute to PD-related cognitive dysfunction.

Consistent with this interpretation, numerous CSF/PET studies in LBD have linked AD co-pathology to memory impairment (Buongiorno et al., 2011; Akhtar et al., 2017), and executive dysfunction (Liu et al., 2015; Lim et al., 2019; Na et al., 2020; Biundo et al., 2021), even at early PD stages (Fiorenzato et al., 2018). Our findings extend this literature by supporting the use of plasma biomarkers, such as p-tau217, as accessible *in vivo* indicators of amyloid- and tau-related mechanisms contributing to PD cognitive impairment. Although evidence specifically evaluating plasma p-tau217 in LBD remains limited, p-tau217 has been associated with global cognition and abstract reasoning in dementia-free older adults (Sim et al., 2025), while stronger associations with memory dysfunction have been reported in cohorts spanning the full AD spectrum (Fernández Arias et al., 2025).

Furthermore, we assessed specific memory encoding functions with the MoCA memory subtask (MoCA-MIS), whose strongest predictor was a reduced Aβ42/40 ratio, suggesting that amyloid pathology may be particularly relevant to encoding deficits in PD and may act alongside tau-related changes. While our composite memory domain encompassed multiple aspects of memory (immediate, delayed, visuospatial), the MoCA-MIS specifically reflects encoding deficits and has been validated in aging cohorts for identifying individuals with MCI at higher risk of progression to dementia (Kronenberger et al., 2025). Given its association with AD-related pathology, MoCA-MIS may represent a brief and clinically feasible tool to identify PD patients at increased risk of dementia due to concomitant AD co-pathology, potentially reflecting a more “AD-like” amnestic phenotype. In this regard, amyloid PET studies consistently report frequent amyloid accumulation in PDD, contributing to memory deficits and faster cognitive decline (Palermo et al., 2019; Myers et al., 2022).

Beyond memory, a lower Aβ42/40 ratio was also associated with visuospatial and social cognition deficits. Visuospatial impairment is common in PD and may predict progression to dementia. Thus, our results suggest that visuospatial deficits are not driven solely by synuclein pathology but may be exacerbated by AD-related pathology. Indeed, this is supported by evidence linking low plasma Aβ42/40 (Sanchez et al., 2024), and CSF Aβ42 to visuospatial decline (Tufekcioglu et al., 2023; Chen et al., 2026) as well as by PET studies identifying amyloid burden as a key predictor of visuospatial and memory impairments in PD (Myers et al., 2022).

Finally, we identified a novel association between amyloid pathology and socio-cognitive deficits in PD. Social cognition is frequently overlooked in standard PD assessments (Pick et al., 2019; Dodich et al., 2025), despite being included in the DSM-5 criteria, and evidence linking AD pathology to social cognition remains scarce (Chow et al., 2023). These preliminary findings underscore the need for further investigation into the biological basis of socio-cognitive impairment in PD.

In the older adult cohort, p-tau217 significantly predicted subjective cognitive decline (MASCoD) and was associated with poorer global cognition (MoCA and MMSE), memory encoding deficits (MoCA-MIS), and language impairment, supporting its role as an early marker across the AD spectrum. In line with prior evidence, p-tau217 outperformed other biomarkers in detecting early amyloid- and tau-related changes across both preclinical and symptomatic AD stages (Ashton et al., 2022). In addition, MMSE and MoCA performance were also predicted by lower Aβ42/40, reflecting greater amyloid burden, in line with the AD pathological framework in which amyloid and tau act synergistically to drive neurodegeneration.

The Aβ42/40 ratio further emerged as a robust predictor across multiple cognitive domains, including attention, visuospatial abilities, and memory; as observed in other mixed CU/MCI cohorts (Garcia-Escobar et al., 2024). Although associations with MMSE and attention vary, the link to visuospatial function appears more reliable across studies (Pereira et al., 2021; Chen et al., 2022).

Overall, plasma AD biomarkers (p-tau217, and Aβ42/40) were consistently associated with cognitive performance, whereas NfL and GFAP did not track cognitive impairment in either cohort, likely due to limited disease specificity. These findings seem to be supported by the temporal dynamics of AD biomarkers: plasma Aβ42/40 decreases early during amyloid accumulation and plateaus as pathology advances (Ashton et al., 2022), whereas p-tau217 increases proportionally with amyloid burden, initially reflecting amyloid dysmetabolism and later associating with tau pathology (Kaeser et al., 2022)—thus underscoring the clinical utility of p-tau217 in tracking AD progression in longitudinal studies (Mattsson-Carlgren et al., 2021).

### 4.4 Neuropsychiatric symptoms

In PD, depressive symptoms (GDS, BDI-II) were significantly associated with elevated p-tau217 and reduced Aβ42/40, suggesting that mood may also be influenced by underlying AD-related pathological processes. Previous CSF and PET studies have reported similar associations between AD-type pathology and depression in PD (Na et al., 2020; Geng et al., 2024). Likewise, apathy was primarily associated with reduced Aβ42/40, consistent with PET studies linking amyloid burden to apathetic symptoms, possibly through fronto-subcortical reward circuit dysfunction (Zhou et al., 2020; Gasca-Salas et al., 2025). Anxiety symptoms were also significantly predicted by plasma AD biomarkers. Increased p-tau217 correlated with both state (STAI Y-1) and trait anxiety (STAI Y-2). Additionally, Aβ42/40 was associated with trait anxiety. Although these associations require confirmation in future studies, the findings underscore the influence of AD-related pathology on anxiety in PD (Geng et al., 2024).

In contrast, neuropsychiatric symptoms in older adults exhibited a different pattern. Higher plasma NfL levels were linked to more pronounced depressive symptoms (GDS), suggesting that mood disturbances in typical aging may reflect generalized neuroaxonal injury rather than AD-specific pathology. This aligns with prior studies proposing NfL as a marker of behavioral symptoms across the AD continuum (Huang et al., 2024; Xia et al., 2026), although findings remain mixed, with some reporting associations with AD pathology (Miao et al., 2022; Krell-Roesch et al., 2023) and others not (Huang et al., 2024). Of note, no significant associations were observed for anxiety or apathy.

### 4.5 Limitations and strengths

Several limitations should be acknowledged. First, the cross-sectional design precludes assessment of longitudinal trajectories and limits causal inference regarding biomarker-clinical relationships. Second, the sample size, particularly for the PDD subgroup, was small, which may constrain the generalizability of our findings. In addition, given the exploratory nature of the analyses, and the absence of formal correction for multiple comparisons in the regression analyses, these findings should be interpreted cautiously and require confirmation in larger independent cohorts. Third, PET/CSF amyloid measures were not obtained in PD participants to confirm p-tau217 positivity; for this reason, p-tau217 was analyzed as a continuous variable. Of note, p-tau217-positive cases are currently undergoing CSF confirmation, and the older adult cohort underwent amyloid PET.

Despite these limitations, this study has some strengths. We employed a multimodal approach, integrating plasma biomarkers with detailed MRI-derived neurostructural indices, extensive neuropsychological and behavioral assessments. To our knowledge, this is the first study to simultaneously investigate these biomarkers in PD (including p-tau217), providing novel insights into the biological correlates of specific cognitive and behavioral impairments in PD, relative to typical aging/MCI. Additionally, the inclusion of the MASCoD scale allowed for a standardized, multidimensional assessment of subjective cognitive deficits in older adults, enhancing comparability across participants and future studies.

## 5 Conclusion

In PD, p-tau217 was associated with AD-specific MRI changes, global cognition (MoCA), executive and memory dysfunctions, whereas Aβ42/40 was linked to other domain-specific cognitive impairments and reduced functional independence, suggesting that AD-related pathology contributes to cognitive and neuropsychiatric symptoms beyond synuclein pathology.

Altogether, these findings underscore the value of integrating plasma biomarkers with structural MRI and clinical assessments to disentangle overlapping pathologies in PD and typical aging/MCI. A clinical-biological approach may improve early identification of individuals at risk for dementia, enhance prognostic accuracy, and inform clinical trial design. Further longitudinal studies are warranted to clarify the temporal dynamics of these biomarkers across the PD and AD spectrum.

## Data Availability

The raw data supporting the conclusions of this article will be made available by the authors upon reasonable request.

## Footnotes

1
https://ageit.eu/wp/en/

2
https://surfer.nmr.mgh.harvard.edu/
