# Supplementary material for: Distinct neurostructural, cognitive, and neuropsychiatric associations of plasma p-tau217, and Aβ42/40 in Parkinson’s disease and aging cohorts
Source: Front Aging Neurosci. 2026 Jun 10;18:1854831. doi: 10.3389/fnagi.2026.1854831 (PMC13290864; doi:10.3389/fnagi.2026.1854831)
Supplement: Supplementary file 1 [file Data_Sheet_1.pdf]

SUPPLEMENTARY MATERIAL

2 SUPPORTING METHODS

2.1 Neuropsychological battery

The II-level neuropsychological assessment evaluates six cognitive domains—memory, executive functions, attention/working memory, visuospatial abilities, language, and social cognition—based on the criteria outlined in the Diagnostic and Statistical Manual of Mental Disorders (DSM-5) and the MDS guidelines (Emre et al., 2007; APA, 2012; Litvan et al., 2012). Memory was assessed using the Prose Memory Test, and the Rey Auditory Verbal Learning Test (RAVLT) and the delayed copy of Rey–Osterrieth Complex Figure (ROCF) (Gasparini et al., 2024). Executive functions were evaluated through the Stroop Test (Caffarra et al., 2002b), Clock Drawing Test (Siciliano et al., 2016), as well as the phonemic verbal fluency task (Costa et al., 2014). Attention/working memory were assessed using the Trail Making Test (B-A) (Giovagnoli et al., 1996), and the Symbol Digit Modality Test (SDMT, verbal version) (Nocentini et al., 2006), and the alternate verbal fluency task (Costa et al., 2014). Visuospatial abilities were measured by the Judgment of Line Orientation (Benton JLO) (Gullett et al., 2013), and the immediate copy of ROCF (Caffarra et al., 2002a). Language was assessed through category verbal fluency (Costa et al., 2014), and the Boston Naming Test (Williams et al., 1989). Social cognition was evaluated using the Story-Based Empathy Task (Dodich et al., 2015), and the FACE test (Terruzzi et al., 2023).

2.4 Neuroimaging data

Structural MRI data included structural images - T1-weighted 3D gradient-echo Turbo-Field-Echo (isotropic voxel size 1 mm3 isotropic, TR=6.9 ms, TE=3.2 ms, Flip Angle 8°, CS-sense acceleration factor 3.5, acquisition matrix 240x240, 181 sagittal slices) and T2-weighted 3D FLAIR (isotropic voxel size 1.12 mm3, TR=8000 ms, TE=360 ms, TI=2400ms, CS-sense acceleration factor 8, acquisition matrix 220x220, 163 sagittal slices) acquired on a 3T Philips Ingenia scanner equipped with a 32-channel phase-array coil. Head motion was minimized through a head restraint system incorporating foam padding around the subject's head.

Structural MRI data were pre-processed using the default automated cortical reconstruction pipeline implemented in FreeSurfer (v6.0.0; https://surfer.nmr.mgh.harvard.edu/). This protocol includes intensity normalization, inter-modality motion correction, skull stripping, transformation to Talairach space, segmentation of cortical and subcortical grey matter (GM) and white matter (WM) structures, tessellation of the GM–WM boundary, and topology correction (Fischl et al., 2002). Quality control was performed using Freeview to verify the accuracy of FreeSurfer segmentations. A trained technician visually inspected each segmentation slice by slice to ensure reliable cortical thickness estimation.

Misplacement of the pial surface, including errors involving the meninges and skull, was manually corrected for all subjects.

For all participants, three MRI neurostructural indices were obtained: global brain atrophy (Orellana et al., 2016), hippocampal volume as segmented by FreeSurfer, and an AD-specific MRI score as detailed below.

To control for head size and age, hippocampal volumes were adjusted using linear regression with total intracranial volume (eTIV) and age as covariates. The final general hippocampal volume was calculated by averaging these adjusted estimates across both hemispheres. The global brain atrophy index, defined as the proportion of the intracranial space occupied by CSF, was included to account for global structural changes in addition to regional hippocampal atrophy.

The MRI-derived AD score, obtained from a machine-learning model, ranges from 0 to 1, with higher values indicating a greater likelihood of an AD diagnosis (Syaifullah et al., 2021). The underlying algorithm predicts AD based on structural information from whole-brain regions as well as the hippocampus. The MRI AD-score was calculated using a support vector machine trained on the Alzheimer's Disease Neuroimaging Initiative (ADNI) dataset (Shiino et al., 2021; Syaifullah et al., 2021), and in the BAAD software (Ishida et al., 2021). This model demonstrates approximately 90% accuracy in identifying amyloid-β PET-positive individuals with MCI.

Notably, the BAAD software integrates algorithmic analyses from the voxel-based specific regional analysis system for Alzheimer's disease (VSRAD). VSRAD focuses on regions of interest within medial temporal structures—including the entorhinal cortex, hippocampus, and amygdala—where atrophy is commonly observed in AD.

3 SUPPORTING RESULTS

The results off multiple linear regression controlling for H&Y are presented below.

3.1 Association between Plasma Biomarkers and Neurostructural Indices

In the PD cohort, multiple linear regression showed that higher plasma p-tau217 levels were associated with a greater MRI AD-signature (β=0.26, t=2.00, p=0.050). In contrast, no significant associations were observed between the other plasma biomarkers (NfL, GFAP, or Aβ42/40 ratio) and the other neurostructural indices, including global brain atrophy and hippocampal volume.

3.2 Association between Plasma Biomarkers and Functional and Clinical Measures

The associations between plasma biomarkers and functional measures in PD are presented in Figure 2B. Lower plasma Aβ42/40 ratios were significantly associated with greater functional

cognitive impairment, as assessed by the PD-CFRS (β=–0.30, t=–2.59, p=0.012), and with reduced functional independence on the IADL scale (β=0.29, t=2.26, p=0.028).

3.3 Association between Plasma Biomarkers and Cognitive Performance

Regarding global cognition, higher p-tau217 levels were associated with lower MoCA scores (β=–0.27, t=–2.09, p=0.041), while a lower Aβ42/40 ratio was associated with poorer performance on its memory index (MoCA-MIS; β=0.29, t=2.27, p=0.027). No associations were found between plasma biomarkers and MMSE scores.

Analysis of cognitive domains (expressed as z-scores) revealed a negative association between p-tau217 levels and executive functions (β=–0.34, t=–2.68, p=0.010); in addition, higher p-tau217 levels were associated with increase memory deficits (β=–0.27, t=–2.09, p=0.041). Conversely, a lower Aβ42/40 ratio was linked to worse visuospatial abilities (β=0.36, t=3.00, p=0.004), language (β=0.27, t=2.20, p=0.032), and poorer socio-cognitive abilities (β=0.26, t=2.16, p=0.035).

Association between Plasma Biomarkers and Neuropsychiatric Symptoms

Higher p-tau217 levels were significantly associated with increased depressive symptoms, as assessed by the GDS (β=0.35, t=2.45, p=0.018) and the BDI-II scale [higher p-tau217 (β=0.23, t=2.04, p=0.047) and lower Aβ42/40 ratio (β=–0.36, t=–3.23, p=0.002)]. Apathic symptoms were also primarily associated with a lower Aβ42/40 ratio (β=–0.31, t=–2.65, p=0.010).

Regarding anxiety, higher state anxiety was associated with elevated p-tau217 (β=0.37, t=2.82, p=0.007), whereas trait anxiety was primarily predicted by higher p-tau217 (β=0.32, t=2.57, p=0.013) and a lower Aβ42/40 ratio (β=–0.30, t=–2.57, p=0.013).

Table S1. Spearman partial correlations between plasma biomarkers, adjusted for age. Log-transformed values of plasma biomarkers were used, followed by False Discovery Rate (FDR) multiple comparison corrections.

**Parkinson's disease cohort (N = 58)**					
		**GFAP (ng/L)**	**NfL(ng/L)**	**Aβ42/40 (ng/L)**	**p-tau217 (ng/L)**
**GFAP (ng/L)**	r_S	—			
	p_{FDR}	—			
**NfL (ng/L)**	r_S	0.29	—		
	p_{FDR}	0.093	—		
**Aβ42/40 (ng/L)**	r_S	0.04	-0.06	—	
	p_{FDR}	0.759	0.759	—	
**p-tau217 (ng/L)**	r_S	**0.35**	0.18	-0.18	—
	p_{FDR}	0.042	0.276	0.276	—
**Older adult cohort (N = 76)**					
		**GFAP (ng/L)**	**NfL(ng/L)**	**Aβ42/40 (ng/L)**	**p-tau217 (ng/L)**
**GFAP (ng/L)**	r_S	—			
	p_{FDR}	—			
**NfL (ng/L)**	r_S	**0.41**	—		
	p_{FDR}	<0.001	—		
**Aβ42/40 (ng/L)**	r_S	0.01	-0.21	—	
	p_{FDR}	0.950	0.1035	—	
**p-tau217 (ng/L)**	r_S	**0.32**	**0.36**	0.01	—
	p_{FDR}	0.010	0.006	0.950	—

REFERENCES

APA (2012). *Diagnostic and Statistical Manual DSM 5*. Washington: American Psychiatric Pub.

Caffarra, P., Vezzadini, G., Dieci, F., Zonato, F., and Venneri, A. (2002a). Rey-Osterrieth complex figure: normative values in an Italian population sample. *Neurological Sciences* 22, 443–447.

Caffarra, P., Vezzadini, G., Dieci, F., Zonato, F., and Venneri, A. (2002b). Una versione abbreviata del test di Stroop: dati normativi nella popolazione italiana. *Nuova Rivista di Neurologia* 12, 111–115.

Costa, A., Bagoj, E., Monaco, M., Zabberoni, S., De Rosa, S., Papantonio, A. M., et al. (2014). Standardization and normative data obtained in the Italian population for a new verbal fluency instrument, the phonemic/semantic alternate fluency test. *Neurol Sci* 35, 365–372. doi: 10.1007/s10072-013-1520-8

Dodich, A., Cerami, C., Canessa, N., Crespi, C., Iannaccone, S., Marcone, A., et al. (2015). A novel task assessing intention and emotion attribution: Italian standardization and normative data of the Story-based Empathy Task. *Neurol Sci* 36, 1907–1912. doi: 10.1007/s10072-015-2281-3

Emre, M., Aarsland, D., Brown, R., Burn, D. J., Duyckaerts, C., Mizuno, Y., et al. (2007). Clinical diagnostic criteria for dementia associated with Parkinson's disease. *Mov Disord* 22, 1689–707; quiz 1837. doi: 10.1002/mds.21507

Gasparini, M., Scandola, M., Amato, S., Salati, E., Facci, E., Gobbetto, V., et al. (2024). Normative data beyond the total scores: a process score analysis of the Rey's 15 word test in healthy aging and Alzheimer's Disease. *Neurol Sci* 45, 2605–2613. doi: 10.1007/s10072-024-07330-0

Giovagnoli, A. R., Del Pesce, M., Mascheroni, S., Simoncelli, M., Laiacona, M., and Capitani, E. (1996). Trail making test: normative values from 287 normal adult controls. *Ital J Neurol Sci* 17, 305–9.

Gullett, J. M., Price, C. C., Nguyen, P., Okun, M. S., Bauer, R. M., and Bowers, D. (2013). Reliability of three Benton Judgment of Line Orientation short forms in idiopathic Parkinson's disease. *The Clinical Neuropsychologist* 27, 1167–1178.

Litvan, I., Goldman, J. G., Tröster, A. I., Schmand, B. A., Weintraub, D., Petersen, R. C., et al. (2012). Diagnostic criteria for mild cognitive impairment in Parkinson's disease: Movement Disorder Society Task Force guidelines. *Movement Disorders* 27, 349–356. doi: 10.1002/mds.24893

Nocentini, U., Giordano, A., Di Vincenzo, S., Panella, M., and Pasqualetti, P. (2006). The Symbol Digit Modalities Test - Oral version: Italian normative data. *Funct Neurol* 21, 93–96.

Siciliano, M., Santangelo, G., D'Iorio, A., Basile, G., Piscopo, F., Grossi, D., et al. (2016). Rouleau version of the Clock Drawing Test: age- and education-adjusted normative data from a wide Italian sample. *Clin Neuropsychol* 30, 1501–1516. doi: 10.1080/13854046.2016.1241893

Terruzzi, S., Funghi, G., Meli, C., Barozzi, N., Zappini, F., Papagno, C., et al. (2023). The FACE test: a new neuropsychological task to assess the recognition of complex mental states from faces. *Neurol Sci*. doi: 10.1007/s10072-023-06697-w

Williams, B. W., Mack, W., and Henderson, V. W. (1989). Boston Naming Test in Alzheimer's disease. *Neuropsychologia* 27, 1073–1079. doi: 10.1016/0028-3932(89)90186-3
